# Performance Evaluation of Polymeric Tubular Membranes for Wastewater from Rubber Production

**DOI:** 10.3390/membranes16030082

**Published:** 2026-02-25

**Authors:** Sławomir Kempa, Mariola Rajca

**Affiliations:** Department of Water and Wastewater Engineering, Faculty of Energy and Environmental Engineering, Silesian University of Technology, 44-100 Gliwice, Poland

**Keywords:** polymeric tubular membranes, NIS, COD, rubber wastewater, membranes MWCO, PES membrane, PVDF membrane, polymeric membranes fouling

## Abstract

The purpose of this study was to assess the suitability of tubular polymeric ultrafiltration membranes for use in a closed-loop water system within a rubber manufacturing plant. This research focused on determining the transport and separation properties of polymeric tubular membranes during the ultrafiltration of wastewater generated from washing vulcanised rubber hoses. The tests were conducted using the installation of the UF-1 membrane supplied by APEKO Sp. z o.o. This study evaluated the performance of modified PES membranes with a molecular weight cut-off (MWCO) of 4 kDa and PVDF membranes with MWCO of 100 kDa in the wastewater treatment process, as well as the effectiveness of membrane regeneration. Given the characteristics of wastewater, the key parameters for evaluating ultrafiltration performance included the determination of contaminant separation coefficients (R, %) for non-ionic surfactants (NIS) and chemical oxygen demand (COD), as well as turbidity reduction. The results demonstrated that the tested membranes substantially improved the visual quality of the wastewater by reducing turbidity by more than 95% and exhibited high separation efficiency for the analysed contaminants, with initial values of R_NIS_ = 95% and R_COD_ = 85% at the beginning of the ultrafiltration cycle, decreasing to R_NIS_ < 10% and R_COD_ < 10% after several hours of operation. During closed-loop filtration, when a twentyfold concentration of contaminants in the retentate was reached, membrane fouling occurred, significantly reducing filtration performance. Chemical cleaning enabled the recovery of approximately 70% of the initial performance for modified PES membranes and 60% for PVDF membranes.

## 1. Introduction

The recovery and reuse of process water in industry currently represents one of the key challenges related to environmental protection and the optimisation of production costs. In the rubber industry, water consumption is substantial, and wastewater from rubber processing plants contains high levels of dispersed contaminants, toxic compounds, and organic pollutants characterised by COD (Chemical Oxygen Demand) and BOD (Biological Oxygen Demand) parameters, as well as surface-active substances [1]. The specific composition of these effluents is the result of the production technology, in which rubber products are vulcanised using steam. This process involves heating the rubber mixture in closed moulds or autoclaves with steam, enabling effective crosslinking of rubber molecules under high temperature and humidity conditions. However, the presence of steam introduces additional challenges related to rubber adhesion to moulds and the protection of production equipment. Steam, as a heating medium, can increase the risk of rubber compound sticking to mould surfaces and contribute to the formation of deposits and corrosion. To mitigate these issues, specialised anti-adhesive agents are applied, which meet the specific requirements of processes carried out under steam conditions [2,3]. The use of anti-adhesive agents in steam vulcanisation is essential to achieve high-quality rubber products and to maintain production efficiency. Commonly used agents such as talc, silica powder, silicone emulsions, wax emulsions, or fluoropolymer-based formulations must be carefully adapted to specific process conditions, including temperature, humidity, and the intense action of steam [4]. The appropriate selection and application of these agents not only prevent rubber adhesion to moulds but also extend the life of the mould, increase production efficiency, and reduce maintenance-related costs. Nevertheless, these agents, indispensable for proper and efficient vulcanisation, introduce an undesirable environmental impact through the release of toxic substances into aquatic environments. Therefore, increasing research efforts are directed toward the development of environmentally friendly anti-adhesive formulations [5,6].

Traditional wastewater treatment methods used in the rubber industry may be insufficient to achieve the water quality required for reuse in production processes. Consequently, modern membrane technologies are gaining importance as effective separation solutions that enable water recovery. In particular, tubular ultrafiltration membranes are widely applied in challenging industrial environments due to their high resistance to fouling and ease of cleaning [7]. Ultrafiltration (UF) is an advanced membrane separation technique that enables the efficient removal of suspended solids, macromolecules, and organic contaminants from aqueous solutions. Due to these properties, ultrafiltration has found broad application in various sectors, including water treatment, wastewater purification, and the food and pharmaceutical industries [8]. However, in industrial applications using filtration membranes, the phenomenon of biofouling, microbial colonisation of membrane surfaces, often occurs and poses a significant problem in membrane systems. Biofouling requires frequent chemical cleaning, which shortens the life of the membrane and reduces the quality of the product. To mitigate this phenomenon, biocidal additives are incorporated into membrane materials [9,10].

In the context of the rubber industry, ultrafiltration employing polymeric tubular membranes may play a key role in optimising wastewater treatment processes. Reuse of treated effluents within an industrial facility enables the implementation of a closed-loop water system, thereby reducing the demand for fresh water and limiting the volume of wastewater discharged, according to the principles of sustainable development. The introduction of this technology allows compliance with stringent environmental regulations while providing economic benefits through reduced water procurement and wastewater disposal costs. Depending on the degree of effluent contamination, such systems—for example, those applied in the food industry—can achieve recovery rates that exceed 90% [11,12].

The stability of filtration membranes under extreme pH conditions remains one of the key challenges in applications related to the treatment of wastewater containing heavy metals, resource recovery, and process streams in the mining, battery recycling, and semiconductor industries. Conventional thin-film composite (TFC) membranes based on polyamide exhibit a limited range of stable operation, typically between pH 4 and 10, and undergo degradation under strongly acidic or alkaline conditions due to hydrolysis of amide bonds or nucleophilic attack on carbonyl groups, leading to a decline in selectivity and structural damage. To address these limitations, alternative material systems for selective layers are being developed, including polysulfonamides, polyamines, and polyureas, which aim to provide enhanced chemical resistance while maintaining a favourable balance between permeability and salt rejection.

Recent studies indicate that selective layers composed of polyurea can effectively combine high durability in strongly acidic and alkaline environments with excellent process performance. Xue et al. presented thin-film capillary membranes in which a polyurea nanofiltration layer, formed via interfacial polymerisation between PEI (polyethyleneimine) and TDI (toluene diisocyanate), achieved a water permeance of 16.8 L/m^2^·h with a rejection of MgCl_2_ of 94.4%, surpassing previously reported flat-sheet polyurea membranes. Importantly, these membranes maintained high MgCl_2_ rejection (91.6–93.9%) after 45 days of continuous exposure to both acidic (pH 1.5) and strongly alkaline (pH 13) solutions, confirming their exceptional chemical stability under extreme pH conditions. The combination of a capillary architecture and a chemically resistant polyurea layer thus establishes a promising platform for designing filtration membranes intended for long-term operation in aggressive industrial environments [13].

However, the topic of wastewater treatment in rubber manufacturing remains relatively under-represented in the available scientific literature and is still considered a novel research field. Up to 2010, no more than ten articles per year addressed this subject, increasing to approximately fifteen in 2015. After 2020, the number of related publications increased significantly to around forty to fifty per year. In particular, nearly 48% of all publications focused on this topic have been released within the past five years [14], indicating that it is a rapidly developing research area with strong potential for further exploration.

Polymeric tubular membranes that show potential for application in the treatment of industrial wastewater from rubber production include, for example, PES membranes and PVDF membranes. Such membranes made of modified PES are widely applied in various industrial sectors, including aerospace, medicine (e.g., in the production of haemolysis philtres) and water treatment and wastewater purification [15,16]. One of the key limitations of ultrafiltration processes with the use of PES membranes is fouling, which leads to reduced process efficiency due to membrane blockage. To mitigate this effect, various material modifications of PES have been developed [17,18]. As a result of these modifications, PES membranes, particularly in tubular form, exhibit excellent filtration performance, high resistance to fouling, and a long operational lifetime, suggesting that they may serve as an optimal solution for wastewater purification in rubber production processes.

PVDF membranes are characterised by high chemical and thermal resistance, which enables their use in various industrial conditions, including processes involving aggressive contaminants and elevated temperatures [19,20]. Furthermore, through simple surface modification techniques, PVDF membranes can be endowed with hydrophilic and antifouling properties, effectively reducing the deposition of contaminants and significantly extending the philtre life [21,22].

The aim of the present study was to evaluate the applicability of the ultrafiltration process employing polymeric tubular membranes for implementation within a closed-loop water system at a manufacturing facility located in southern Poland, which specialises in the production of rubber components for the automotive industry. The intended application is designed to allow reuse of the process water originating from the washing of vulcanised rubber hoses, which is currently discharged into the sewage system. The process would include preliminary treatment and water recovery for subsequent reuse in production operations. Particular attention was paid to parameters such as the concentration of non-ionic surfactants (NISs), the chemical oxygen demand (COD), and the turbidity, which represent the primary contaminants in this category of industrial wastewater. The reduction of these parameters serves as an indicator of the efficiency of process water purification.

## 2. Materials and Methods

### 2.1. Tested Wastewater

The tested wastewater came from the washing process of the vulcanised rubber hoses prior to their final assembly stage. The washing is carried out using warm water at 45 °C without the addition of detergents. The main source of contamination in this wastewater comprises surfactants derived from an anti-adhesive lubricant used during the steam vulcanisation of rubber. This lubricant originates from the vulcanisation stage that precedes the washing process. It adheres to rubber hoses when steam autoclave operators apply it to steel moulding fittings to facilitate hose installation. This operation ensures smooth assembly, prevents mechanical damage, and avoids adhesion of the hoses to the fittings. The lubricant used in this process, Rheolase 487 LG, is highly soluble in water and fully dissolves at temperatures above 45 °C [23].

A high chemical oxygen demand (COD) value is characteristic of such organic anti-adhesive agents, which contain substances that are readily oxidised in aqueous environments, including fats, oils, waxes and water-soluble organic compounds [24].

The wastewater samples used in this study were collected from a drainage manhole located in the production hall and transferred to a process tank over consecutive days of testing. The characteristics of the wastewater analysed are presented in Table 1.

### 2.2. Ultrafiltration Process

In the present ultrafiltration studies, an APEKO UF-1 pilot installation was used, incorporating a B1-type tubular membrane module manufactured by PCI Membranes Filtration Group. The B1 module allows for the installation of 18 tubular membranes operating in a cross-flow configuration. Due to their tubular design, such membranes are particularly suitable for filtering liquids with high viscosity and those containing significant amounts of suspended solids because of their wide flow channels offering high resistance to blockage and enabling the effective separation of solutions containing solid particles [25,26,27]. In addition, the membrane surfaces were protected against biofouling by applying a biocidal agent. The impregnation agent used was Proxel GXL, supplied by Azelis Essential Chemicals (Berchem, Belgium), a water-based biocide containing 20% dipropylene glycol.

In the study, two types of tubular membranes were used. The first type was ESP04 membranes manufactured by PCI Membranes, made of modified polyethersulphone (PES).

The second type of tubular membrane used in this study was FP100 membranes, also manufactured by PCI Membranes, made of polyvinylidene fluoride (PVDF). These membranes were characterised by a higher molecular weight cut-off value (MWCO) compared to the modified PES membranes, indicating the potential for achieving significantly greater process efficiency. The technical parameters of the tested membranes are presented in Table 2.

### 2.3. Research Installation

**Figure 1:**
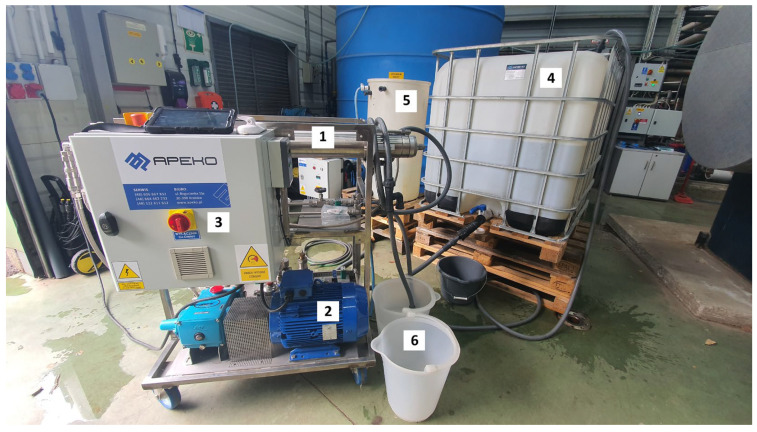
View of the APEKO UF-1 pilot-scale installation. 1—Cylindrical PCI B1 module equipped with 18 membrane elements; 2—CAT high-pressure recirculation pump; 3—Electrical control box with operating panel; 4—1000 L IBC tank containing wastewater feed for ultrafiltration; 5—Cleaning chemical tank; 6—Reference permeate container.

**Figure 2:**
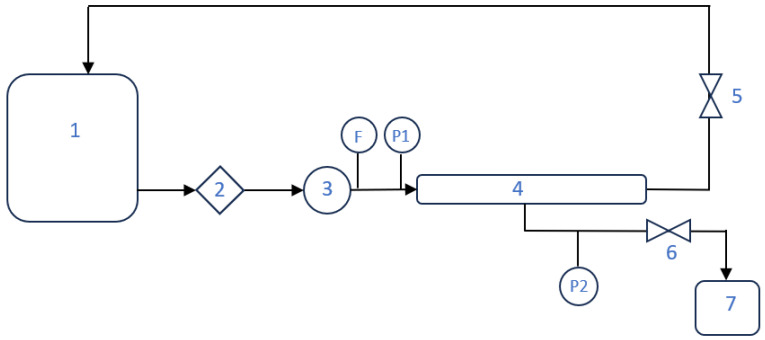
Flow diagram of the pilot-scale APEKO UF-1 installation. 1—Wastewater feed tank; 2—50 µm string wound pre-philtre; 3—Pump; 4—B1 membrane module; 5—Retentate valve; 6—Permeate valve; 7—Permeate tank; P1—Feed pressure gauge; P2—Permeate pressure gauge; F—Feed flowmeter.

The experiments were conducted on an APEKO-supplied UF-1 pilot device, equipped with a B1 membrane module and PCI Membranes tubular membranes. The photograph of the installation is shown in Figure 1, and its process flow diagram is presented in Figure 2. The nominal performance of the installation, determined for new membranes using clean water, is presented in Table 3 (softened municipal tap water was used for this purpose).

### 2.4. Parameters Analysed

In this study, the transport and separation conditions of the membranes were determined during the ultrafiltration of successive wastewater batches. Changes in volumetric permeate flux were examined as a function of time and volume of filtered wastewater. The permeate flux was calculated using the following equation:

(1) $$J=\frac{V}{F\times t}$$

*J*—volumetric permeate flux [L/m^2^·h]; *V*—volume of permeate obtained over time *t* [L]; *F*—effective membrane area [m^2^] *t*—time [h].

The nominal permeate flux (for pure water) for the tested membrane module with ESP04 membranes was:

(2) $$Jn=\frac{3.33\;L}{0.9\;m^{2}\times 0.01667\;h}=221.95\;\frac{L}{m^{2}\times h}$$

The nominal permeate flux (for pure water) for the tested membrane module with FP100 membranes was:

(3) $$Jn=\frac{11.43\;L}{0.9\;m^{2}\times 0.01667\;h}=761.84\;\frac{L}{m^{2}\times h}$$

The contaminant separation coefficient for the tested membranes was calculated using the following formula:

(4) $$R=1-\frac{C_{p}}{C_{n}}\times 100$$

*R*—pollutant separation coefficient, *C_p_*—pollutant concentration in the permeate, *C_n_*—pollutant concentration in the wastewater (feed).

During this study, the parameters of the raw wastewater and the permeate obtained were determined, including the chemical oxygen demand (COD), the concentration of non-ionic surfactants (NIS), the specific conductivity, the organic and inorganic carbon content, and the turbidity during ultrafiltration of successive batches of wastewater. Parameter analyses were performed using a Hach Lange Sp. z o.o. (Wrocław, Poland) DR9000 spectrophotometer and cuvette tests LCI400 (COD analysis) and LCK433 (non-ionic surfactant analysis), supplied by Hach Lange Sp. z o.o. Poland. The tests were performed according to the Hach procedures [28,29]. Turbidity was measured using an Engineered Systems & Designs, Model 800 turbidimeter, Newark, NJ, USA. Organic and inorganic carbon contents were measured using a TOC-L series analyser (Shimadzu, Tokyo, Japan). The zeta potential for membranes was determined using the Surpass electrokinetic analyser by Anton Paar (Graz, Austria). Measurements were conducted using KCL (0.01 M) as the primary electrolyte, and changes in pH during titration were made by adding solutions of HCl or NaOH (0.1 M). SEM images were taken using a high-resolution SUPRA 35 scanning electron microscope from ZEISS (Oberkochen, Germany). Secondary electron (SE) detection at an accelerating voltage of 20 kV and maximum magnifications of up to 200,000× were used to acquire the images. The chemical composition analysis of the samples was performed using an energy-dispersive X-ray spectroscopy (EDS) spectrometer TRIDENT XM4 from EDAX (Mahwah, NJ, USA). The zeta potential of the wastewater was determined using a Zetasizer Z (Malvern Panalytical, Malvern, UK). The total solids content (dry mass) was determined by drying the raw wastewater at a temperature of 105 °C.

### 2.5. Research Methodology

The experiments were conducted at a rubber manufacturing and processing plant located in Southern Poland, where a pilot-scale installation of APEKO UF-1 (Apeko, Kraków, Poland) equipped with a PCI Membranes B1 module (PCI Membranes Filtration Group, Fareham, UK),was installed directly on the production floor. Wastewater was collected from a sewer inspection chamber serving the rubber washing machines into a 1 m^3^ plastic IBC tank and subsequently subjected to ultrafiltration. To evaluate the performance of the membrane system tested, successive tests were carried out while maintaining a constant transmembrane pressure and a constant feed flow rate of 23 L/min. For the ESP04 membranes (PCI Membranes, Fareham, UK), the wastewater feed volume was 600 L, and the initial transmembrane feed pressure was 15 bar, which was increased to 20 bar during the experiments and to 25 bar in the final trial to compensate for flux decline. For the FP100 membranes (PCI Membranes, Fareham, UK), which were expected to provide higher throughput, the wastewater feed volume was 1000 L. In these tests, the feed flow rate was also maintained at 23 L/min, while the feed transmembrane pressure remained constant at 8 bar throughout the filtration process.

The experiments were conducted in a closed batch configuration under concentration mode with constant TMP, in which a defined volume of wastewater in the IBC tank was subjected to ultrafiltration. The retentate was continuously recirculated to the feed tank, while the permeate was collected simultaneously. Such an operating mode led to a gradual increase in the concentration of contaminants retained by the membrane in the feed tank and to a progressive deterioration of process conditions on the membrane surface. The process was continued until fouling occurred, resulting in membrane blockage and a sharp decline in its performance. This made it possible to determine the critical concentration at which the membrane lost its operational efficiency and to evaluate the effectiveness of fouling removal by chemical cleaning.

During each ultrafiltration trial, permeate and retentate samples were collected at hourly intervals. For each batch of wastewater (WW1–WW3), a total of ten samples were obtained, including five permeate samples and five retentate samples. The samples were collected in 200 mL glass containers, labelled with the corresponding date and time, and immediately stored at 4 °C until analysis. All physicochemical analyses were performed on the last day of operation for the first membrane (ESP04) and subsequently following the same procedure for the second membrane (FP100).

After each filtration cycle (each wastewater batch WW1–WW6), the membranes were subjected to chemical cleaning, followed by performance verification through filtration with softened, clean water. This procedure was intended to assess the cleaning effectiveness and regeneration capability of the membranes, thereby minimising the effects of fouling.

Membrane regeneration was carried out through a chemical cleaning process using two Ecolab products from the Ultrasil series: P3 Ultrasil 02 and P3 Ultrasil 11. P3 Ultrasil 02 is an alkaline liquid detergent based on surfactants and sequestrants, while P3 Ultrasil 11 is an acidic cleaning agent used as a complement to the alkaline cleaning with P3 Ultrasil 02. The alkaline agent is designed to remove organic contaminants, whereas the acidic agent eliminates mineral residues. The membrane cleaning solution was prepared in accordance with the manufacturer’s recommendations. A mixture of 150 g of the alkaline agent, Ultrasil 11, and 30 mL of the acidic agent, Ultrasil 02, was dissolved in 50 L of softened tap water, treated on site using the plant’s water conditioning system. The prepared solution was heated to 50 °C and then circulated through the membrane module for one hour under a constant feed pressure of 10 bar for ESP04 membranes and 6 bar for FP100 membranes.

For the FP100 membrane, the zeta potential was additionally determined before the ultrafiltration of wastewater (for a new membrane) and after the completion of the process. This measurement was carried out to assess changes in the membrane potential during operation and to examine how the zeta potential could affect the filtration efficiency of wastewater containing surfactants and contaminants expressed as chemical oxygen demand (COD). In addition, the zeta potential of the wastewater was determined, and the relationship between zeta potential and membrane fouling was subsequently analysed, as fouling is one of the key factors limiting the performance of the ultrafiltration process.

## 3. Results and Discussion

**Figure 3:**
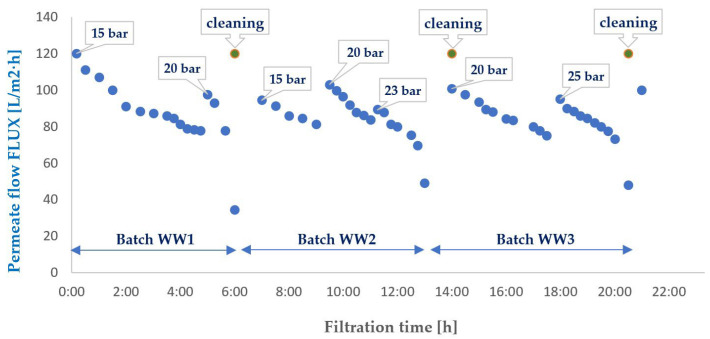
Variation in the volumetric permeate flux during ultrafiltration of wastewater. The results were normalised to a temperature of 20 °C. ESP04 membrane.

**Figure 4:**
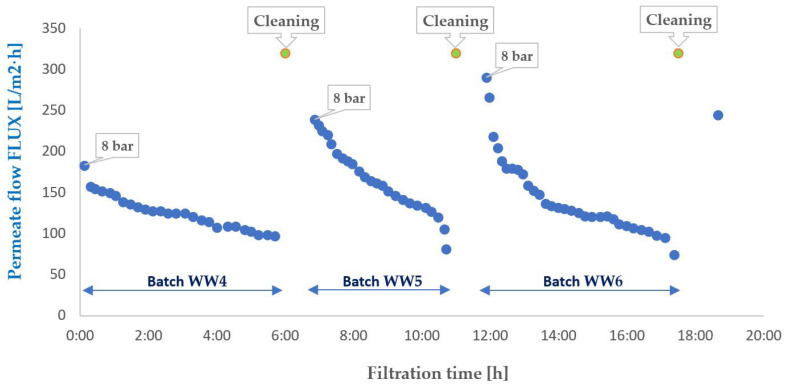
Variation in the volumetric permeate flux during ultrafiltration of wastewater. The results were normalised to a temperature of 20 °C. FP100 membrane.

**Figure 5:**
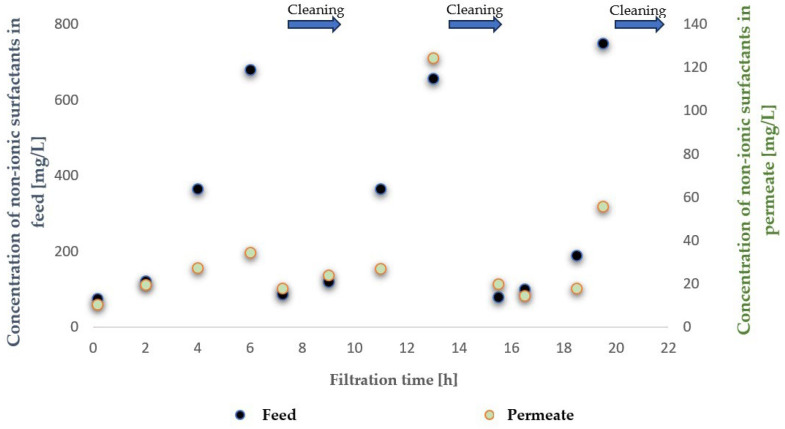
Contents of non-ionic surfactants in the feed and permeate during ultrafiltration using the ESP04 membrane.

**Figure 6:**
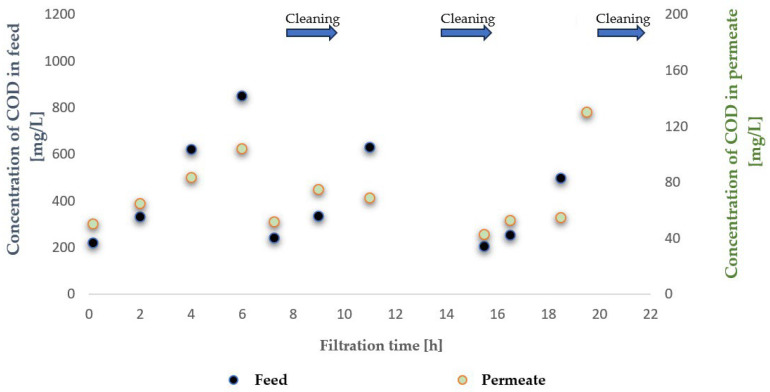
Contents of contaminants expressed as chemical oxygen demand (COD) in the feed and permeate during ultrafiltration using the ESP04 membrane.

**Figure 7:**
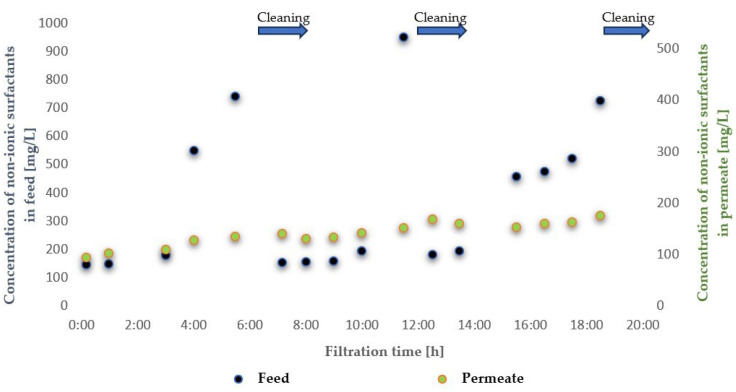
Contents of non-ionic surfactants in the feed and permeate during ultrafiltration using the FP100 membrane.

**Figure 8:**
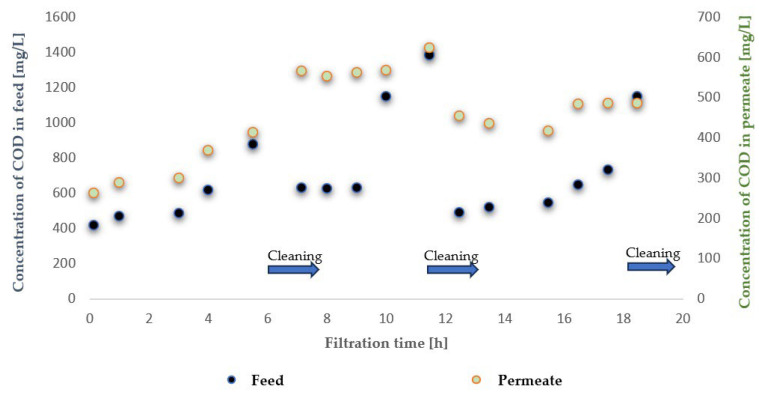
Contents of contaminants expressed as COD in the feed and permeate during ultrafiltration using the FP100 membrane.

**Figure 9:**
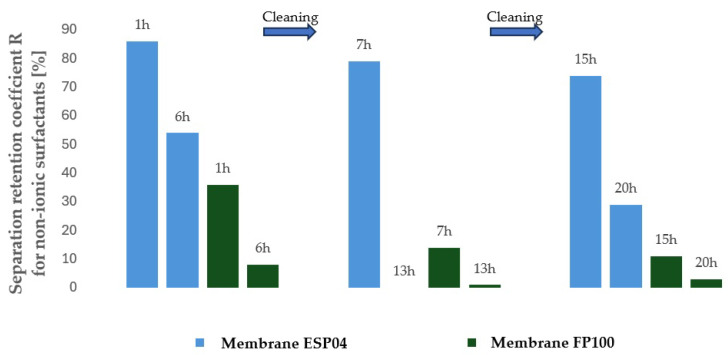
Retention coefficient of non-ionic surfactants for ultrafiltration using ESP04 and FP100 membranes.

**Figure 10:**
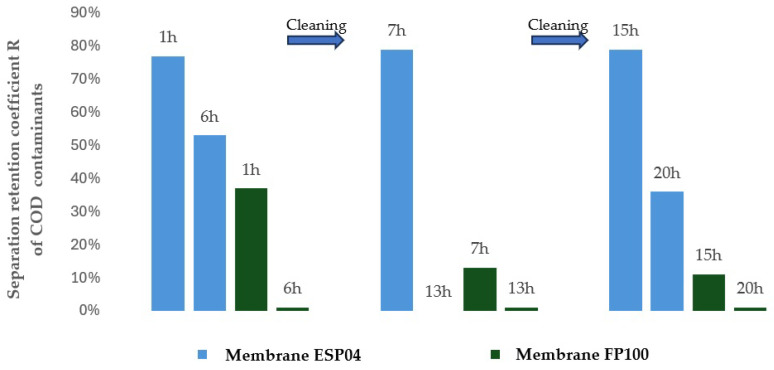
Separation coefficient for COD contaminants during ultrafiltration using ESP04 and FP100 membranes.

**Figure 11:**
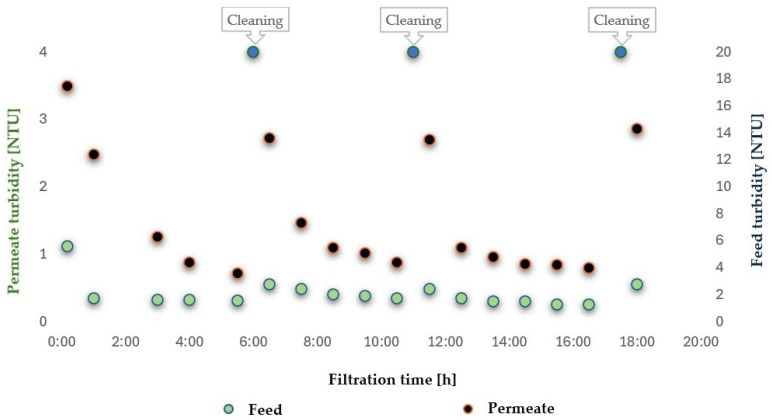
Turbidity of feed and permeate for ultrafiltration of wastewater using FP100 membranes.

**Figure 12:**
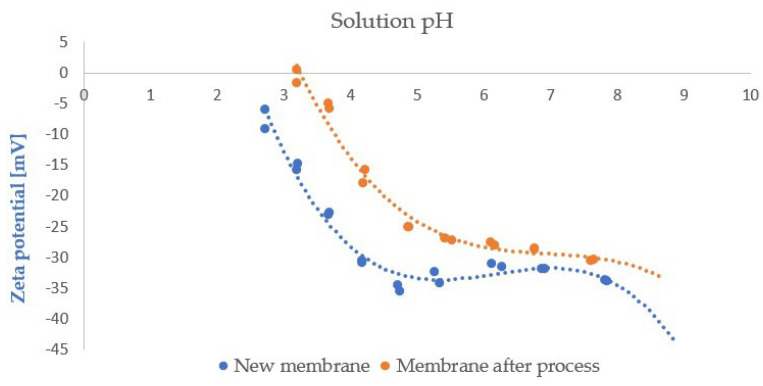
Zeta potential for new FP100 membrane and after wastewater ultrafiltration process.

To evaluate the suitability of the tested membranes for the intended application, the experiment was conducted in a closed-loop system with retentate recirculation and progressive concentration of the separated contaminants, an approach commonly referred to in the literature as the batch method, which enables a comprehensive assessment of the applicability of selected membranes to a given type of wastewater [30,31,32]. During the experiments, membrane transport conditions were monitored (Figure 3 and Figure 4) by analysing variations in volumetric permeate flux, which indicates membrane performance, over time with constant TMP and feed flow rate. To evaluate the efficiency and suitability of the process for the intended applications, the separation conditions were analysed for key contaminants. These included the concentration of non-ionic surfactants (NISs), the chemical oxygen demand (COD), the turbidity, as well as the content of total, inorganic and organic carbon (TOC, IC and TC) (Figure 5, Figure 6, Figure 7, Figure 8, Figure 9, Figure 10, Figure 11 and Figure 12).

The first three cycles, which included wastewater batches from WW1 to WW3, were conducted with installed ESP 04 PCI Membranes. The selection of these membranes was justified by their numerous advantages that make them suitable for the intended application. This type of membrane has been applied, among others, in studies by Woźniak P. and Gryta M. in car wash effluents [33], where their good separation properties were confirmed for contaminants of similar nature, such as a high surfactant content, considerable concentrations of organic pollutants expressed as COD, and waxes.

Investigations using FP100 membranes were carried out for wastewater batches from WW4 to WW6. As expected, a higher performance was obtained for the FP100 membranes, although its magnitude was slightly lower than expected. The mean values of the volumetric permeate flux J, along with its initial and final values, are presented in Table 4.

In comparative studies of polymeric tubular ultrafiltration membranes FP100 and ESP04, significant differences in filtration performance were observed, arising from both inherent membrane properties and operational conditions in which they were used. FP100 membranes, possessing a molecular weight cut-off (MWCO) of 100 kDa, were characterised by larger pore sizes, which translated into higher permeability and superior fouling resistance compared to ESP04 membranes with a substantially lower MWCO of 4 kDa. Consequently, FP100 membranes exhibited greater permeate flux during the process, consistent with literature observations indicating that higher MWCO contributes to reduced pore clogging risk and enables easier flow [33,34].

The studies were conducted in a batch system with retentate recirculation to the feed and permeate collection. In practice, this meant that the pollutant concentration in the feed increased over time, exerting a significant influence on filtration parameters and fouling dynamics. During FP100 membrane operation, a constant feed flow and a stable transmembrane pressure of 8 bar were maintained, allowing for a linear, gradual decline in permeate flux (Figure 4) for WW4 and WW5 batches. The evolution of the volumetric permeate flux for the WW6 wastewater feed is initially steeper over approximately the first hour, after which it transitions to a more moderate linear trend. This linear performance drop, consistent with the fouling models described in the literature [35], enables the planning of regular regeneration cycles, minimising the risk of sudden adverse events.

For the ESP04 membranes, which are characterised by very small pores (MWCO 4 kDa), the process was initiated at a pressure of 15 bar, corresponding to half of the maximum pressure specified for this membrane by the manufacturer. The initial flux decline for these membranes also proceeded in a linear manner (Figure 3). During the experiments, stepwise increases in transmembrane pressure were applied, first to 20 bar and subsequently to 25 bar, in an attempt to counteract the flux decline. Although the pressure increase transiently boosted permeate flow (Figure 3), it simultaneously accelerated membrane fouling, leading to the rapid deterioration of operational parameters and limited effective operating time. This aligns with literature descriptions that, for membranes with lower MWCO, pressure elevation can convert the fouling cake layer into a more compact and less permeable structure, restricting flow [36,37].

In the ultrafiltration experiments of rubber-production wastewater characterized by very high chemical oxygen demand (COD) and a strong tendency to cause organic fouling, the application of a transmembrane pressure (TMP) as high as 15–25 bar is associated with a significant risk of membrane compaction. Compaction involves mechanical compression of the polymer matrix, a reduction in porosity, and a decrease in the effective pore size, which together lead to a progressive and partly irreversible loss of permeability between subsequent filtration cycles, irrespective of fouling caused by the feed components themselves. Previous studies on pressure-driven polymeric membranes have demonstrated that, even in the range of approximately 10–40 bar, a marked reduction in porosity and water permeability can occur due to structural densification of the support and selective layers, which supports the assumption that similar or more severe deformation may develop under the conditions applied in the present work [38].

The observation that, after each chemical cleaning step, the membrane recovered only about 70% of its initial water flux suggests a substantial contribution of irreversible phenomena, including both persistent organic fouling and permanent structural changes resulting from compaction. Long-term ultrafiltration studies have shown that, even under efficient cleaning protocols, the maximum achievable flux of polymeric membranes tends to stabilize at values considerably below the nominal initial permeability, which is commonly attributed to a combination of non-removable fouling and mechanically induced changes in membrane structure. In the present case, the batch operating mode with volume reduction and concentration factors up to approximately 20 further intensified these effects, since the increasing concentration of macromolecules, surfactants, and latex at the membrane surface promoted the formation of a highly compressed cake layer that, under high TMP, could be pressed into the pores and co-deformed with the membrane matrix [39].

From an interpretative standpoint, the fact that the membrane maintained good rejection of contaminants and relatively stable performance up to a concentration factor of about 20 does not exclude gradual deterioration of its intrinsic structure due to compaction. The observed decline in permeate flux in the final stage of concentration is, therefore, the combined result of concentration effects and fouling, compression of the deposited layer, and progressive compaction of the membrane itself, which is not fully reversible, even after intensive chemical cleaning. Consequently, any further operation of this membrane at lower TMP should be evaluated with the understanding that its hydraulic and separation properties no longer correspond to those of the pristine material, and that the measured fluxes reflect a permanently compacted structure with reduced porosity [40].

In the closed-loop installation configuration used in this study, as shown in Figure 2, the rubber production wastewater is continuously recirculated from tank 1 through a 50 µm string filter (2), a pump (3), and the UF module (4). As a result, each portion of the stream passes several times through the prefilter before being concentrated in the retentate or discharged as permeate. From the perspective of mass balance, this means that over time, an increasing fraction of suspended solids larger than 50 µm is mechanically retained in the string filter, leading to a systematic decrease in the overall turbidity of the feed tank. This behaviour is typical of batch-type UF systems with cross-flow filtration supported by mechanical prefiltration, where cartridge-type preliminary elements with nominal ratings of a few to several dozen micrometres capture the bulk of coarse particles, while the membrane module operates primarily on the colloidal and dissolved fractions.

Simultaneously, the turbidity of the permeate remains low and almost constant, below approximately 1 NTU, indicating that the membrane effectively retains finer colloids and other mechanical impurities from the process. Furthermore, cleaning procedures restore membrane separation capacity without significant fluctuations in filtrate quality, consistent with observations reported for UF applied to wastewater and surface water treatment.

Therefore, the decrease in feed turbidity observed in this study, concurrent with an increase in the dissolved pollutant load, is not an unusual phenomenon but rather a consequence of the separation of two contaminant fractions: the suspended fraction, effectively removed by the 50 µm filter, and the dissolved fraction, concentrated in the retentate as the UF process progresses.

In the investigated rubber production wastewater, a high content of non-ionic surfactants and organic compounds expressed as COD was observed. These are largely present in micellar and true solution form rather than as classical suspensions responsible for turbidity. During recirculation and successive permeate withdrawal, the concentration of these components in the retentate increases, increasing both the non-ionic surfactant concentration and the COD load, and consequently the potential for organic membrane fouling, while the concurrent “purification” of the feed of particles > 50 µm in the string filter leads to a systematic reduction in turbidity measured in the feed tank.

With respect to other pollutant parameters, such as chemical oxygen demand (COD) and non-ionic surfactants (NISs), the membranes provided good initial separation of these substances, but the permeate quality deteriorated as pollutant concentrations in the feed increased. This deterioration of the permeate parameters is characteristic of batch systems with pollutant concentration and has been confirmed in other studies, indicating the need to optimise the operation and the cleaning frequency [41,42].

It was observed that after approximately six hours of operation for both types of membranes, a sharp decrease in process performance occurred due to the accumulation of pollutants, leading to pore blockage and increased hydraulic resistance. This phenomenon is frequently described in the literature as a critical point in membrane operations requiring regenerative intervention [43].

A more detailed analysis of the permeate flux curves for the membranes ESP04 and FP100 (Figure 3 and Figure 4) reveals a clear differentiation in fouling dynamics depending on the filtration time and the intrinsic properties of each membrane. During the initial hours of each filtration cycle, the flux of membrane ESP04 decreases from an initial level of approximately 100–120 L/m^2^·h to approximately 70–80 L/m^2^·h, while for membrane FP100, it decreases from around 200–250 L/m^2^·h to roughly 130–150 L/m^2^·h, exhibiting a relatively smooth quasi-linear trend. This type of behaviour corresponds to the classical cake filtration mechanism according to Hermia’s models, where the growing deposit layer on the membrane surface constitutes the dominant component of the overall hydraulic resistance, and the flux decline rate is largely governed by the rate of particle deposition and compression at the interface.

In the literature, a similar shape of flux curve, gradual, nearly linear decline under constant pressure has been associated with the formation of a relatively porous, highly permeable cake layer that can be partially removed by hydrodynamic backwashing under moderate shear conditions. The noticeable repeatability of curve slopes in subsequent cycles after membrane regeneration, in particular, suggests that the dominant and reversible fouling component is indeed the surface-layer deposit. Similar observations have been reported for UF/NF membranes that filter wastewater rich in colloids and organic matter [44].

In the final phase of each cycle, especially immediately before cleaning, the flux curves display a distinct inflexion, with a rapid acceleration of performance decline, and the data points forming a noticeably steeper segment. This effect is particularly evident for membrane ESP04 in all wastewater batches (WW1–WW3), where the flux decreases from approximately 80 to below 40 L/m^2^·h in a relatively short period. For membrane FP100, this phenomenon is not observed for WW4 feed, for which the concentration factor was the lowest (<10). In contrast, for WW5 and WW6, feeds once the concentration factor exceeded 15, the effect became clearly pronounced.

This pattern is consistent with a transition from dominant cake filtration to pore blocking mechanisms (standard blocking or intermediate blocking) according to Hermia’s classification, where finer suspended fractions or dissolved organic compounds of high molecular weight penetrate the pore structure, generating resistance that is less amenable to removal by cleaning. As reported in the literature on UF/NF fouling in the presence of colloid–natural organic matter mixtures, increasing the concentration of solute in the boundary layer leads to compaction of the cake layer, enhanced adhesive forces and a gradual transition from surface adsorption to deeper pore blockage, manifested as the rapid decrease in flux in the final stage of filtration.

The experimental data obtained were quantitatively fitted to the Hermia models: complete blocking, standard blocking, intermediate blocking, and cake filtration, to identify the dominant mechanism for each membrane and process stage, directly following the guidelines established in standard membrane fouling analysis procedures [45].

In the classical form of Hermia’s model, the starting point is a differential equation that relates the volume of the permeate to the filtration time.

The following linearised forms are commonly used:

- Complete blocking (*n* = 2):

$$\frac{1}{J}=\frac{1}{J_{0}}+k\;t$$

- Intermediate blocking (*n* = 1)

$$ln\;(J)=ln\;(J_{0})-k\;t$$

- Standard blocking (*n* = 1.5):

$$\frac{1}{\sqrt{J}}=\frac{1}{\sqrt{J_{0}}}+k\;t$$

- Cake filtration (*n* = 0):

$$\frac{1}{J^{2}}=\frac{1}{J_{0}^{2}}+2k\;t$$

*J*_0_—initial permeate FLUX, *t*—filtration time, *J*—permeate FLUX at time *t*, *k*—the slope coefficient determined experimentally.

After applying the equations to the flux values obtained during the experiments for the ESP04 and FP100 membranes for each subsequent batch of wastewater (WW1–WW6), the corresponding fouling models were fitted. The results are presented in Table 5.

Membrane regeneration allowed only partial recovery of their initial properties. The ESP04 membranes regained approximately 70% of their initial permeate flux (Table 6) when normalised to a temperature of 20 °C, indicating permanent changes induced during the fouling process, typical of membranes with smaller pores and complex surface morphology [46]. It should be noted, however, that flux loss did not accumulate over successive cycles, and the average temperature-normalised flux at 20 °C remained nearly identical for each wastewater feed (WW1–WW3), reaching 85 L/m^2^·h (Table 4). Statistical analysis using ANOVA, performed for the permeate flux data of the ESP04 membrane (results presented in Table 7), showed no statistically significant differences in flux values, which may be interpreted as consistent and stable membrane performance across all wastewater batches (WW1–WW3).

In the case of FP100 membranes, the regeneration process also partially mitigated fouling and enabled the recovery of approximately 60% of the initial flux (Table 5). However, the average permeate flux during wastewater filtration obtained for these membranes was higher than for ESP04 membranes, exceeding 120 L/m^2^·h in each trial. The data for consecutive wastewater batches (WW4–WW6) are presented in Table 4. For the obtained permeate flux values of this membrane, the ANOVA analysis indicated statistically significant differences, suggesting a lack of reproducibility of membrane performance between wastewater batches. This lack of reproducibility may result from variations in the composition of the wastewater in successive batches or from changes in the predominant fouling mechanism.

It should be emphasised that although FP100 membranes demonstrated higher overall flux performance and similar fouling resistance, significant differences were observed in the separation of dissolved organic contaminants. ESP04 membranes, having a lower MWCO = 4 kDa, exhibited a superior removal of chemical oxygen demand (COD) and non-ionic surfactants (NIS). Practically, this indicated that the ESP04 membranes more effectively separated these dissolved organic substances from wastewater compared to the FP100 membranes, which showed only minor rejection of NIS and negligible reduction in COD, as illustrated in Figure 10 and Figure 11. This aligns with the literature indicating that membranes with smaller pores, i.e., lower MWCO, retain smaller organic molecules and dissolved substances more effectively, albeit at the expense of reduced permeability and increased fouling [47,48].

In summary, studies conducted and the literature review indicate that membrane pore size (MWCO) exerts a significant influence on the efficiency of the ultrafiltration process, fouling mechanisms, and regeneration capabilities. The batch system with retentate recirculation induces an increase in contaminant concentration in the feed, which requires consideration in the design and operation of installations, particularly with respect to membrane selection and cleaning strategies. Proper management of these factors enables effective wastewater treatment by maintaining high permeate quality and long-term performance of the membrane system [49,50].

**Figure 13:**
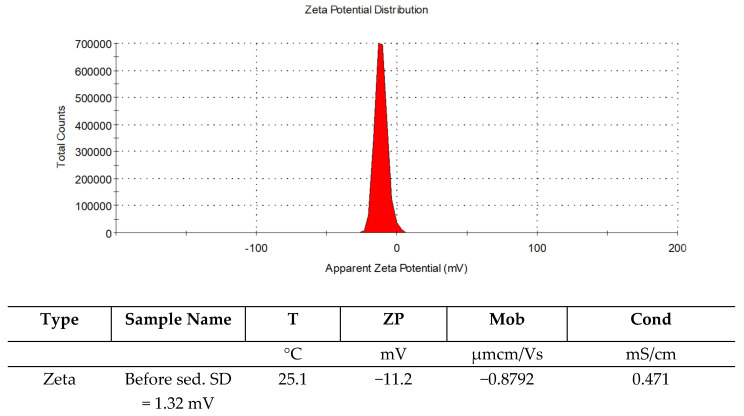
Zeta potential of filtered wastewater.

To identify the fouling characteristics of the FP100 membrane, which demonstrated higher process performance than the ESP04 membrane, its surface was analysed using scanning electron microscopy (SEM) and zeta potential measurements before and after filtration. The zeta potential results for the new and used membranes are presented in Figure 12. The zeta potential of the wastewater samples was also measured, and the result is presented in Figure 13. The new membrane exhibited a negative zeta potential throughout the investigated pH range. Within the pH range 7 to 8, relevant to the application conditions of the process, the potential values ranged between −35 and −30 mV. After filtration, a shift in the zeta potential towards more positive values was observed throughout the pH range, particularly pronounced at pH < 5. At higher pH values, these differences were less significant.

The results indicate that the new membrane surface was strongly negatively charged at higher pH values, approaching the isoelectric point at lower pH values. The most negative values (~−35 mV) occurred within pH 4.7–7.8, typical for polymeric ultrafiltration membranes. As the pH decreased below 4, a marked reduction in negative charge was observed (approximately −6 to −15 mV), suggesting protonation of surface groups due to the increase in the concentration of H^+^ ions [51,52].

The data obtained indicate that, at low pH values, the membrane exhibits increased susceptibility to adsorption of positively charged contaminants, whereas under alkaline conditions, its resistance to fouling by anionic colloids and organic substances increases. A strongly negative zeta potential promotes electrostatic repulsion of similarly charged particles (anions, colloids, humics), limiting their adsorption and deposition on the surface. This is particularly advantageous in the analysis process, as the wastewater subjected to ultrafiltration had a pH in the range of 7.5 to 7.9. Thus, a highly negative zeta potential may contribute to reducing the rate of irreversible accumulation of fouling, facilitating its removal during rinsing, and allowing stable membrane operation under neutral or slightly alkaline conditions [53,54].

The wastewater sample exhibited a zeta potential of −11.2 mV, i.e., a negative value, which is consistent with typical values reported for suspensions in wastewater and surface waters. The negative charge of the wastewater promotes electrostatic repulsion between the negatively charged membrane and the majority of colloids and organic fractions present in the solution, limiting their irreversible adsorption. Under neutral to slightly alkaline conditions (pH 7.5–7.9), corresponding to the installation operating conditions, the strongly negative surface charge of the FP100 membrane can therefore slow the development of irreversible fouling and facilitate its removal during hydraulic cleaning [55].

The results obtained indicate that in low-pH environments, the membrane shows an increased tendency to adsorb positively charged ions and complexes (e.g., Ca^2+^, Mg^2+^) and to form mineral deposits, leading to inorganic fouling. Under alkaline conditions, its resistance to fouling induced by anionic colloids and organic substances is greater; however, the observed decrease in the absolute value of the zeta potential after filtration suggests a gradual reduction of this protective effect [56].

Non-ionic surfactants present in wastewater also play an important role in the development of fouling. Despite the absence of an electrical charge in their polar head groups, these compounds can adsorb strongly onto the membrane surface, primarily due to hydrophobic interactions between the alkyl chains of the surfactants and the more hydrophobic domains of the polymer, as well as other hydrophobic constituents of the wastewater. The adsorbed layers formed in this way are capable of screening the charge of ionic groups at the surface and reducing the absolute value of the zeta potential, even in the absence of direct electrostatic interactions. In addition, the non-ionic surfactant layer facilitates the anchoring of additional foulants (including hydrophobic organic fractions, colloidal particles, and polymers), thereby enhancing organic fouling and contributing to a permanent modification of the properties of the membrane surface [57].

The observed shifts in zeta potential after filtration clearly confirm the occurrence of fouling involving the adsorption of both ionic and non-ionic species (including surfactants), leading to a change in the surface character of the membrane. These findings highlight the need for regular membrane regeneration or chemical cleaning to remove mineral scale layers and surfactant films and thus restore the original hydrophilicity, a strongly negative zeta potential, and a favourable separation performance [58].

**Figure 14:**
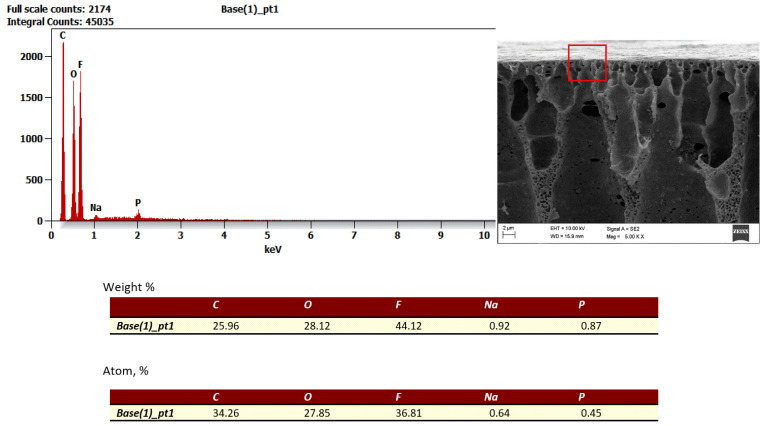
SEM results for a new FP100 membrane.

**Figure 15:**
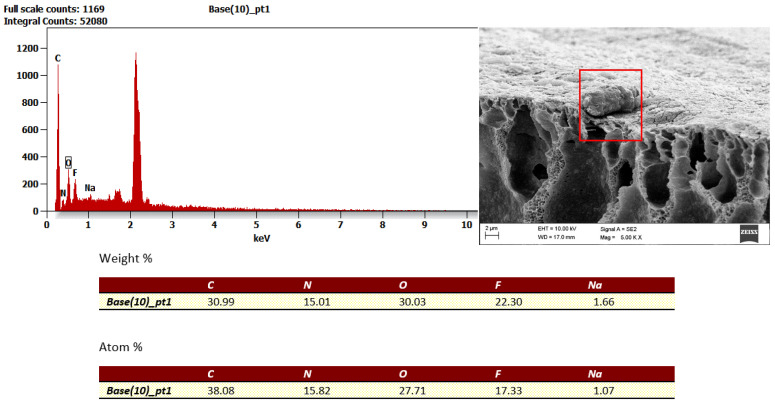
SEM results for FP100 membrane after ultrafiltration process.

To confirm the nature of fouling and changes in zeta potential, a detailed analysis of the membrane surface and pore structure was performed using electron beam imaging, i.e., SEM examination. The results for the new FP100 membrane are presented in Figure 14, while those for the FP100 membrane after the wastewater ultrafiltration process are shown in Figure 15.

The SEM image analysis of the PVDF polymeric filtration membrane with a nominal MWCO of 100 kDa, obtained at magnifications ranging from 63× to 176×, allowed for a detailed assessment of its microstructure and porosity. The surface of the membrane exhibits a distinctly porous structure with pores of varying sizes and shapes. Areas of increased material density are also noticeable, indicating the heterogeneous nature of the membrane structure. Local accumulations of deposits and contaminants are visible on the surface, indicating the presence of fouling, including potential oily deposits. In some areas, morphological changes, such as cracks or deformations of the membrane layer, were observed, likely resulting from membrane operation and suboptimal conditions.

The porous structure of the examined membrane aligns with its filtration purpose, where the appropriate porosity and pore distribution are crucial to achieve effective contaminant separation while ensuring efficient medium flow. Observations of fouling and local deposits highlight the need to optimise cleaning procedures to restore the original filtration properties of the membrane. In addition, detected mechanical defects and surface changes can adversely affect the durability of the membrane, requiring systematic diagnostics of operating conditions and potential adjustments to the process parameters.

The dominance of fluorine and carbon confirms the use of PVDF as the membrane construction material, while the presence of aluminium, silicon, sodium, sulphur, and magnesium indicates the operation under industrial wastewater conditions that favour the formation of complex mineral–organic deposits. The diversity and irregular distribution of these contaminants suggest the need for an individualised approach to membrane cleaning and regeneration, aimed at the effective removal of both mineral deposits (Al, Si, and Mg) and organic compounds, as well as fluorine-containing species.

The presence of metal and alkaline earth ions also affects the electrokinetic properties of the PVDF membrane. Sodium (Na) may modulate the surface electrostatic charge, shifting the zeta potential towards less negative values. Aluminium (Al) forms hydroxyl complexes interacting with the membrane surface, whereas silicone (Si), typically present in the form of silicates, alters the local chemistry and electrostatic interactions. Sulphur (S), probably occurring as sulphates, and magnesium (Mg), capable of complexing with functional groups, further influence the zeta potential. Consequently, the observed changes in potential during the operation arise from the presence of these ions, which modify the surface charge, fouling mechanisms, and the reversibility of cleaning processes.

The wastewater used in this study originated from the rubber manufacturing industry, in which sodium, sulphur, aluminium, and silicon naturally occur. These are characteristic elements derived from mineral fillers, auxiliary agents, and technological processes such as vulcanisation and thermal treatment. Consequently, these elements are present in process water and wastewater, which must be taken into account when planning cleaning and regeneration strategies for membranes operated in this type of industrial installation [1,59].

For the membrane materials investigated (PES and PVDF), the dominant signals of C, O, and F can be mainly attributed to their polymeric structure. A thin fouling layer may partially mask elements typical of wastewater (Na, Al, Si, Mg, S), whose contents are detected only at trace levels. Nevertheless, their presence indicates co-adsorption of colloids and mineral particles characteristic of wastewater from the rubber industry, confirming the complex composition of the developing boundary layer [57].

The formation mechanism of this layer is primarily associated with the synergistic action of concentration polarisation and the adsorption of non-ionic surfactant micelles within the pores and on the membrane surface. As the concentration of surfactant in the wastewater increases, the critical micelle concentration (CMC) is locally exceeded, which promotes the formation of aggregates with dimensions retained by the PES/PVDF membranes. Intensive adsorption of micelles and co-occurring organic matter leads to a gradual decline in permeate flux (Figure 3 and Figure 4), while part of the deposit remains strongly bound to the surface, confirming a significant contribution of irreversible adsorption [60].

Changes in the zeta potential of the FP100 membrane after ultrafiltration indicate a shift of the curve towards less negative values over the entire pH range, which unambiguously confirms surface coverage by a layer of surfactants and organic substances that modify the structure of the electrical double layer. The literature indicates that under such conditions, the zeta potential of the system increasingly depends on the properties of the fouling layer rather than on the original membrane material, resulting in a decrease in the absolute value of the potential and a shift of the isoelectric point.

An integrated interpretation of the SEM/EDS results, zeta potential measurements, and flux characteristics confirms that the main factor that limits the performance of the ultrafiltration process is adsorption–polarisation fouling, induced by micellar forms of nonionic surfactants, coadsorbed organic matter, and mineral particles present in industrial wastewater.

Studies in the literature confirm that the primary fouling mechanisms of PVDF ultrafiltration membranes are pore blocking and cake layer formation, driven by the adsorption of organic substances, minerals and metal ions present in wastewater. Optimisation of membrane operation and cleaning processes should account for the specificity of the contaminant and their impact on electrokinetic property changes to effectively minimise fouling and extend the life of the membrane system [61,62].

Considering the application of ultrafiltration using tested polymeric tubular membranes to establish a closed water loop in the washing of vulcanised rubber hoses, this method proves highly suitable for improving the visual parameters of wastewater by significantly reducing turbidity (Figure 12). Additionally, low MWCO membranes, such as the ESP04 tested with 4 kDa, exhibit good separation efficiency for contaminants defined as COD. In contrast, higher MWCO membranes such as the FP100 tested with 100 kDa showed ineffective separation of dissolved surfactants and other organics. In the intended target application, namely water recovery for the washing of rubber hoses, the parameters of the obtained permeate must meet the requirements specified in Table 8.

A comparison of the permeate parameters obtained for both membranes indicates that, in terms of pH, turbidity, and electrical conductivity, both membranes produce permeate of satisfactory quality. Regarding non-ionic detergents and dissolved organic compounds, expressed as COD, quality requirements are met for WW1–WW3 wastewater feeds filtered through ESP04 membranes. In contrast, for WW4–WW6 wastewater feeds filtered through FP100 membranes, these criteria are not met, which limits their suitability for the application considered.

Furthermore, analysis of permeate flux data and fouling models showed that, for the ESP04 membranes, the flux is more stable and reproducible, whereas the FP100 membranes exhibit a change in the fouling model for feed WW5, as well as statistically significant differences in flux between subsequent feeds, as confirmed by ANOVA analysis.

An important parameter from the perspective of practical operation is also the behaviour of the membranes after successive regeneration cycles: as shown in Table 4, the ESP04 membranes exhibit a gradual decrease in flux loss with successive feeds, and the average flux values in individual tests remain comparable. In contrast, for FP100 membranes, the average flux values differ between tests and the flux loss after each subsequent regeneration cycle increases (Table 4). Consequently, from the point of view of practical application, ESP04 membranes, due to their more stable permeate flux and greater operational stability, appear to represent a more advantageous choice for the application examined [63,64].

In summary, when assessing the feasibility of applying ESP04 membranes in a closed water circulation system to wash rubber hoses, the quantitative balance of recovered water is of key importance. For WW1–WW6 feed batches, high recovery values were obtained, as presented in Table 9, which confirms the technical potential of reintroducing the ultrafiltration permeate into the process loop.

Similar levels of water recovery (above 90%) have been reported in the literature for ultrafiltration systems applied to industrial wastewater, where UF serves as a key step in water recovery schemes and in reducing the consumption of fresh water resources. For the preferred ESP04 membrane, a recovery rate of approximately 95% indicates a significant potential for direct recirculation of water back into the production process. This means that only a small fraction of the volumetric stream remains as concentrate, which is typical for modern systems designed to achieve high recovery rates and minimise wastewater generation.

However, such a high degree of water recovery inevitably results in a considerable enrichment of contaminants in the retentate; at 95% recovery, concentration increases of roughly 20-fold compared to the raw wastewater can be expected. Concentrates of this type require targeted management following the approaches discussed in studies devoted to the handling of membrane system concentrates and the Zero Liquid Discharge (ZLD) concept [65].

An important component of the proposed solution is the possibility of directing the concentrated retentate to the existing evaporation unit in the plant, which is currently used to treat wastewater generated during the rubber vulcanisation process. The literature indicates that integration of membrane processes (MF/UF, NF/RO) with vacuum evaporators or conventional evaporation units represents one of the most effective strategies for concentrate management, allowing for further increases in water recovery and for minimising the volume of liquid waste. Incorporating ultrafiltration retentate in the underloaded evaporator at the analysed rubber plant could allow an overall water recovery of approximately 98–99%, in line with the ZLD concept and with the trends reported in studies on industrial water reuse and recovery [66].

## 4. Conclusions

Key final conclusions from the ultrafiltration membrane studies (FP100 and ESP04) on wastewater from washing vulcanised rubber hoses are as follows:

1. The FP100 membranes (MWCO) exhibited higher performance, with the average permeate flux being 40–50% greater than that of the ESP04 membranes; however, they showed lower stability, as the flux was not consistently reproducible during successive wastewater feed runs.
2. ESP04 membranes (MWCO 4 kDa) achieved superior reductions in chemical oxygen demand (COD) and non-ionic surfactants (NIS), while FP100 membranes showed minimal separation of NIS and negligible reduction of COD.
3. Both membranes significantly reduced wastewater turbidity, confirming their effectiveness in removing suspensions and colloids.
4. Increasing contaminant concentration in the feed due to retentate recirculation in the batch system led to increased fouling and gradual deterioration of filtration parameters over time.
5. After approximately 6 h of operation, a sharp decline in permeate flux required membrane regeneration.
6. Membrane regeneration enabled the recovery of approximately 70% of the initial flux for the ESP04 membranes and about 60% for the FP100 membranes.
7. The strongly negative zeta potential of the new FP100 membrane (−30 to −35 mV at pH values from 7 to 8 together with negative zeta potential for wastewater (−11 mV), typical for wastewater from the rubber industry at pH 7.5–7.9, provides high fouling resistance against anionic colloids and organic substances via electrostatic repulsion. However, in the presence of non-ionic surfactant contaminants, the primary fouling mechanism is the adsorption of particles onto the hydrophobic surface.
8. After filtration, the zeta potential shifted towards positive values (particularly at pH < 5) due to adsorption of organic contaminants, colloids and surfactants. SEM and EDS analyses confirmed deposits (Al, Si, Na, S, Mg at 1–3%) and local accumulations, indicating the need for regular regeneration to restore hydrophilicity and separation properties.
9. The permeate obtained from the FP100 membranes did not meet the quality requirements for water recovery for process reuse. In contrast, the permeate generated using the ESP04 membranes is suitable for reuse in the production process.
10. Ultrafiltration employing the ESP04 membranes for this wastewater enables water recovery of approximately 95%.

## Figures and Tables

**Table 1:** Parameters of wastewater collected for testing in subsequent batches.

Parameter	COD [mg/L]	NIS [mg/L]	Turbidity [NTU]	Conductivity [µS/cm]	pH	TDS [mg/L]	TOC [mg/L]	TC [mg/L]	IC [mg/L]	Dry Mass [%]
WW1 *	219.0	73.9	51.20	287	7.8	184	98.5	126.1	27.6	0.036
WW2	240.0	86.5	50.30	295	7.7	189	108.0	135.5	27.3	0.033
WW3	204.0	78.1	48.70	282	7.9	180	91.8	119.6	27.8	0.035
WW4	418.0	145.0	52.30	331	7.9	212	94.3	121.9	27.6	0.038
WW5	630.0	151.0	38.40	195	7.9	125	139.3	166.5	27.2	0.044
WW6	488.0	180.0	70.30	318	7.3	203	133.7	162.2	28.5	0.041

* WW1–WW6—subsequent batches of wastewater collected for testing.

**Table 2:** Parameters of PCI tubular membranes used in the study.

Membrane	MWCO	Material	Diameter	pH	Max TMP	Max T
ESP04	4 kDa	PES	12.5 mm	1–14	30 bar	65 °C
FP100	100 kDa	PVDF	12.5 mm	1.5–12	10 bar	80 °C

**Table 3:** Initial membranes efficiency.

Membrane’s Type	Quantity of Tubes	Membrane’s Surface	TMP	* Initial Flow
ESP04	18	0.9 m^2^	4.0 bar	222 L/m^2^/h
FP100	18	0.9 m^2^	4.0 bar	762 L/m^2^/h

* The pure water flux obtained for the virgin membrane at the beginning of the experiment.

**Table 4:** Values of the average permeate flux, as well as the initial and final fluxes for each test, normalised to the standard temperature of 20 °C.

Wastewater Sample	Membrane Type	Average Flux at 20 °C [L/m^2^·h]	Initial Flux [L/m^2^·h]	Initial Flux at 20 °C [L/m^2^·h]	Final Flux [L/m^2^·h]	Final Flux at 20 °C [L/m^2^·h]	Loss of Flux [%]
WW1	ESP04	82	160 (33 °C)	108	49 (37 °C)	34	69%
WW2	ESP04	85	118 (34 °C)	94	73 (42 °C)	45	52%
WW3	ESP04	85	116 (31 °C)	100	100 (40 °C)	73	27%
WW4	FP100	126	251 (34 °C)	183	140 (37 °C)	96	48%
WW5	FP100	166	327 (34 °C)	239	117 (38 °C)	81	66%
WW6	FP100	146	404 (35 °C)	290	104 (35 °C)	74	74%

**Table 5:** Matching of the Hermia fouling models.

Membrane Type	Wastewater Batch	TMP [bar]	Fouling Type	Matching Factor R^2^
ESP04	WW1	15	Cake filtration	0.979
	WW1	20	Intermediate blocking	0.967
	WW2	15	Cake filtration	0.977
	WW2	20	Cake filtration	0.989
	WW2	23	Intermediate blocking	0.961
	WW3	20	Cake filtration	0.985
	WW3	25	Standard blocking	0.973
FP100	WW4	8	Cake filtration	0.975
	WW5	8	Intermediate blocking	0.983
	WW6	8	Cake filtration a	0.978

**Table 6:** Comparison of permeate fluxes for clean water, normalised to a temperature of 20 °C, after successive regeneration cycles.

Membrane	Flux 20 °C	Loss of Flux
ESP new	221	-
ESP After 1 cleaning	189	14%
ESP after 2 cleanings	141	36%
ESP after 3 cleanings	158	29%
FP100 new	764	-
FP100 After 1 cleaning	441	42%
FP100 After 2 cleanings	472	38%
FP100 After 3 cleanings	453	41%

**Table 7:** Results of ANOVA variance analysis for the measured permeate flux values obtained in successive experimental runs.

Membrane	*p*-Value	F	Significance of Flux Differences Between Samples
ESP04	0.7516	0.2871	Statistically non-significant differences
FP100	0.0024	6.5378	Statistically significant differences

**Table 8:** Water quality parameters for the hose-washing process.

Parameter	Value
pH	6.5–8.5
Conductivity	<500 µS
Turbidity	<1 NTU
Non-ionic surfactant concentration	<50 mg/L O_2_
COD concentration	<500 mg/L O_2_

**Table 9:** Comparison of the achieved water recovery efficiency for membranes.

Membrane Type	Wastewater Sample	Feed Volume [L]	Permeate Volume [L]	Concentrate Volume [L]	Recovered Water [%]
ESP04	WW1	600	510	90	85%
ESP04	WW2	600	570	30	95%
ESP04	WW3	600	570	30	95%
FP100	WW4	950	780	170	82%
FP100	WW5	800	720	80	90%
FP100	WW6	1000	930	70	93%

## Data Availability

The original contributions presented in this study are included in the article. Further inquiries can be directed to the corresponding authors.
